# Mark My Words: Tone of Voice Changes Affective Word Representations in Memory

**DOI:** 10.1371/journal.pone.0009080

**Published:** 2010-02-15

**Authors:** Annett Schirmer

**Affiliations:** Department of Psychology, National University of Singapore, Singapore, Singapore; University of Groningen, Netherlands

## Abstract

The present study explored the effect of speaker prosody on the representation of words in memory. To this end, participants were presented with a series of words and asked to remember the words for a subsequent recognition test. During study, words were presented auditorily with an emotional or neutral prosody, whereas during test, words were presented visually. Recognition performance was comparable for words studied with emotional and neutral prosody. However, subsequent valence ratings indicated that study prosody changed the affective representation of words in memory. Compared to words with neutral prosody, words with sad prosody were later rated as more negative and words with happy prosody were later rated as more positive. Interestingly, the participants' ability to remember study prosody failed to predict this effect, suggesting that changes in word valence were implicit and associated with initial word processing rather than word retrieval. Taken together these results identify a mechanism by which speakers can have sustained effects on listener attitudes towards word referents.

## Introduction

Spoken language, like other communication systems, evolved as a means for influencing the attitudes and behaviours of communication partners [1]–[3]. That spoken language is particularly powerful in this influence likely has two reasons. First, language is the only biological communication system that is truly generative [4]. Unlike nonverbal messages, which are limited in number and scope, language comprises a set of arbitrary symbols whose combination allows for an infinite number of potentially complex and abstract messages. A second and equally important fact is that language uses as its vehicle the voice–a communication system already present in our pre-linguistic ancestors [5], [6]. Emotion induced bodily changes affect the functioning of the voice thereby modulating the rate, intensity, and spectral quality of vocalizations. These modulations, also referred to as prosody, add emotional significance to a verbal message thereby increasing its persuasive power.

Past research investigated whether and how prosody augments the influence of spoken language on listeners. Of particular interest has been the question whether emotional prosody captures attention more readily than neutral prosody. Behavioral evidence to this effect comes from an investigation of spatial attention [7]. Spatial locations are more effectively cued by emotional as compared to neutral vocalizations. Additionally, neuroimaging research provides evidence. For example, fMRI studies found larger activity in the superior temporal sulcus (STS) for emotional as compared to neutral prosody regardless of whether prosody was task-relevant [8]–[12]. Given the role of the STS in higher order auditory processing, this observation suggests that emotional prosody recruits more processing resources and is thus more likely to be noticed. A similar conclusion was derived from auditory odd-ball studies using event-related potentials (ERPs). In such studies, participants typically perform a foreground task while a task-irrelevant auditory sequence is presented in the background. Rare auditory deviants elicit a mismatch negativity (MMN) indicative of pre-attentive change detection (for a review see [13]). Importantly, this negativity is larger for vocal emotional as compared to neutral deviants, again suggesting that listeners are more likely to notice the former kind of utterance [14], [15].

A second focus of interest in the study of prosody has been the integration of prosodic and verbal information. This has been investigated using both explicit emotion judgments and implicit priming paradigms. Explicit emotion judgment studies typically presented semantically neutral, negative, or positive valence words spoken with neutral, negative, or positive prosody [16]–[19]. Thus, word valence and prosody were emotionally congruous or incongruous. Participants performed word valence judgments faster and more accurately when emotional prosody was congruous as compared to incongruous. Similar results emerged from implicit priming studies. Here participants performed lexical decisions on emotion words whose valence was congruous or incongruous to that of a preceding prosodic prime. Faster lexical decisions were observed for the earlier as compared to the latter condition [20]. Functional neuroimaging evidence suggests that these effects reflect the retrieval of word information from semantic memory [20]–[22]. Accordingly, such retrieval appears to be facilitated for congruous relative to incongruous prosodic and verbal emotions allowing congruous messages to be more easily understood and acted on.

While these immediate effects of prosody on language processing are relatively well established, little is known about potentially sustained effects on listener attitudes and behavior. In particular, one may ask whether prosody influences the representation of a verbal message in long-term memory as that representation will determine whether and how people act on the message. One way such an influence may occur is by enhancing memory for verbal messages that are delivered with an emotional as compared to neutral prosody. Indirect support for this proposition comes from published memory research (for a review see [23]–[25]). Words, like other stimuli, were found to be better remembered when they convey an emotional as compared to neutral meaning [26]–[28]. More importantly, memory for neutral words can be improved when they are embedded in an emotional sentence relative to when they are embedded in a neutral sentence [26]–[28]. Thus, one may conclude that verbal context modulates memory for individual words and speculate that prosody, another form of context, may have a similar effect. This speculation is partially supported by a study on incidental speech processing [29]. In this study, participants engaged in a numeric short-term memory task while passively listening to sentences pronounced with positive, neutral, or negative prosody. Incidental memory for negatively spoken sentences was higher than that for neutral or positively spoken sentences suggesting that negative prosody facilitated the storage of verbal information. However, as this finding was specific to an incidental encoding condition with a high short-term memory load, it is unclear how pervasive the influence of emotional prosody really is and whether it extends to a situation in which speech processing is intentional.

A second way in which prosody could influence the representation of a verbal message in memory is by adjusting its emotional significance or valence. After all, words are just arbitrary combinations of phonemes that derive their valence from what they symbolize, which in turn derives its valence from experience. This experience can be direct through interactions with a word's referent or indirect through communications that relay such interactions. For example, after being bitten by a dog or learning from another individual that dogs bite, the word that represents dogs may come to symbolize threat and acquire a negative valence. Evidence for this comes from classical conditioning research demonstrating that individuals fear symbols that have been paired with an electric shock or for which they have been told that such a shock may occur [30]. In both cases, they respond with increased physiological arousal relative to a symbol for which neither a direct nor an indirect negative experience is available. Given that words are symbols, one may infer that their emotional significance is equally malleable. Moreover, one may speculate that a word's context, such as speaker prosody, continuously modulates word valence.

The present study probed this speculation and investigated whether and how prosody influences the storage of intentionally processed speech. Participants were asked to memorize a series of neutral words spoken with neutral or sad prosody. Subsequently, these words were presented together with new words in a visual word recognition test. In this test, participants indicated whether a word was old or new. Both old and new decisions were followed by a word valence rating for which participants judged each word on a 5 point scale ranging from −2 (very negative) to +2 (very positive). If emotional prosody influences word processing in the ways outlined above, we should observe better word recognition of old words that were studied with sad as compared to neutral prosody. Additionally, old words studied with sad prosody should be rated as more negative than old words studied with neutral prosody.

## Methods

### Experiment 1

#### Participants

Thirty-two undergraduate students participated in the experiment. Half the participants were female with an average age of 21.8 years (SD 2.4). Male participants were on average 22.7 years (SD 1.5). Participants were enrolled in an introductory level psychology module and received course credit for participating. All participants reported normal or corrected to normal vision as well as normal hearing. They signed informed consent prior to the experiment.

#### Materials

A set of 500 words was rated by a group of 30 independent raters (15 female) on two 5-point scales, one ranging from −2 (very negative) to +2 (very positive) for word valence and one ranging from 0 (non-arousing) to 4 (highly arousing) for arousal. Based on these ratings, 240 neutral valence (mean 0.16, SD 0.20), weakly arousing (mean 0.58, SD 0.24) words were selected. Frequency measures (Kucera-Francis Written Frequency: mean 57.2, SD 76.5) were obtained from the MRC Psycholinguistic Database.

The speaker for this and the experiments reported below was selected based on a rating study. For this study, we invited four individuals with drama experience. These individuals were asked to portray the selected 240 words with anger, sadness, happiness and neutrality. All words were recorded and digitized at a 16 bit/44.1 KHz sampling rate. Word amplitude was normalized at the root-mean-square value using Adobe Audition 2.0. A subset of the same 15 words was selected for each prosodic condition and each speaker. These words were presented in random order to a group of 30 listeners (15 female) who were asked to indicate whether the speaker pronouncing a given word was in an angry, sad, happy, or neutral emotional state or in an emotional state not listed (e.g., disgust). They then had to rate each vocalization on a five-point scale from −2 (very negative) to +2 (very positive) with respect to emotional valence and on a five-point scale ranging from 0 (not aroused) to 4 (very aroused) with respect to arousal. For Experiment 1, we selected the speaker who portrayed sadness and neutrality better than all the other speakers. Her rating results are presented in Table 1. The average duration of words produced by this speaker was 1132.4 ms (SD 245.5) for sad prosody and 777.6 ms (SD 149) for neutral prosody.

**Table 1:** Stimulus rating results.

	Identification Accuracy	Emotional Valence	Emotional Arousal
***Experiment 1/3***			
Sad Prosody	88% (14)*	−1.45 (0.45)	2.92 (0.76)
Neutral Prosody	89% (14)	0.06 (0.15)	0.79 (0.61)
***Experiment 2***			
Happy Prosody	85% (15)	1.26 (0.32)	2.73 (0.51)
Neutral Prosody	85% (15)	0.01 (0.14)	0.97 (0.60)

*Standard Deviation in parenthesis.

#### Procedure

Experiment 1 employed a verbal memory paradigm consisting of two blocks with a study phase and a test phase each. A study phase comprised 60 trials. Each trial started with a fixation cross. After 500 ms, a word was presented over headphones while the fixation cross remained on the screen. The fixation cross disappeared at word offset. On half the trials, words were spoken with a sad prosody whereas the remaining trials used neutrally spoken words. The order of trials was randomized and the inter-trial interval (ITI) was 1000 ms. Each study phase was followed by a test phase comprising 120 trials. Again, each trial started with a 500 ms fixation cross. The cross was replaced by a word in the center of the computer screen. On half the trials, the word was from the preceding study phase, whereas on the remaining trials the word was new. Upon reading a word, participants were asked to press one of two buttons indicating whether the word was “old” or “new”. Once participants pressed the appropriate button, the word disappeared from the screen and they were now prompted to rate the valence of the word on a 5-point scale ranging from −2 (very negative) to +2 (very positive). The screen turned black after participants completed the rating and the next trial started after 1000 ms.

The stimulus set of 240 words was split into four sets of 60 words each. These sets were presented as (1) old words with sad prosody, (2) old words with neutral prosody, and (3/4) new words. A given word was presented only once to a given participant. However, across participants, they appeared equally often as old and new words and equally often as words with sad and neutral prosody. Words were rotated in this way to avoid any stimulus confound on the effects of interest. To avoid a dexterity related response confound, we also counterbalanced the assignment of old/new judgments to left and right response buttons.

Prior to the experiment, participants were instructed to listen to the words in each study phase and were informed that their memory for these words would be assessed in a subsequent word recognition test. In order to clear any doubts about the general procedure, participants performed a practice run composed of six study trials followed by 12 test trials using the dummy words from the stimulus recording. Test trials in this practice run comprised old/new decisions only. Participants were informed about the word valence rating only when commencing the word recognition test in the actual experiment.

#### Results

**Figure 1:**
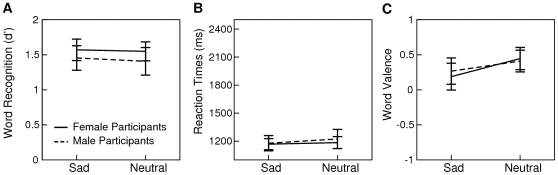
Results from Experiment 1. Mean d' scores and standard errors reflecting the sensitivity of discriminating old from new words are illustrated in graph A. Mean reaction times to correctly recognized old words are illustrated in graph B. Mean valence ratings of correctly recognized old words are illustrated in graph C.

The results of Experiment 1 are illustrated in Figure 1. The uncorrected probability of recognizing an old word as old was 0.77 (SD 0.15) for sad prosody and 0.79 (SD 0.14) for neutral prosody. A signal detection framework was applied to the analysis of the word recognition data. To this end, the probability of false alarms was calculated by dividing the number of new words incorrectly classified as old by the actual number of new words. Please note that this value did not differ as a function of prosody as all new words appeared in written form only. The probability of hits was calculated by dividing the number of correctly recognized old words by the actual number of old words in each prosody condition. Thus, hits differed as a function of prosody. A d' score was calculated by subtracting the normalized probability of false alarms from the normalized probability of hits for each prosody condition. The obtained d' scores were subjected to an ANOVA with *Prosody* as a repeated measures factor and *Sex* as a between subjects factor. This analysis revealed no significant effects (ps>.2). A second ANOVA with reaction times to correctly recognized old words as the dependent variable was performed to assess the speed of memory access as a function of *Prosody* and *Sex*. This analysis was also non-significant (ps>.18).

The effect of speaker prosody on word valence was assessed by subjecting the valence ratings of correctly recognized old words to an ANOVA with *Prosody* as a repeated measures factor and *Sex* as a between subjects factor. This analysis revealed a main effect of *Prosody* (F(1,30) = 8.09, p<.01) indicating that participants evaluated words as more negative, when these words were spoken with sad (mean 0.23, SD 0.74) as compared to neutral prosody during study (mean 0.43, SD 0.62).

#### Discussion

The results of Experiment 1 support the claim that speaker prosody has a sustained influence on listener attitudes and behaviour. Participants rated words as more negative if they had studied these words with sad as compared to neutral prosody. Contrary to expectation, however, Experiment 1 failed to reveal an influence of prosody on the accuracy or speed of word recognition. There are at least two possible reasons for this. First, prior work establishing a relationship between emotion and memory has relied on threat-related and/or highly arousing stimuli [26], [27], [30] (for a review see [24]). Moreover, emotional memory effects have been linked to activation of the sympathetic nervous system and feedback from this system to brain structures implicated in memory consolidation (for a review see [25]). As such, stimuli that are emotional but minimally arousing may not effectively enhance memory. Given that some consider sadness to be a low-arousal emotion [5], the sad prosody used here may not have been appropriate to study emotional memory. Alternatively, however, prosody may be irrelevant for the intentional storage of verbal information. Previous emotional context effects on intentional speech processing were based on a within-stimulus manipulation [26]–[28]. Written words were presented together with other words of emotional or neutral meaning. In the present study, the context was of a different quality than the content. While the former was non-linguistic, the latter was linguistic in nature. Under these conditions transfer of emotional significance may not readily occur.

A second experiment was conducted to probe these possibilities. While this experiment was comparable to the previous one in most respects, it differed in that study prosody was either happy or neutral. Happy prosody was selected because it reflects a high-arousal emotion [5] and thus should induce arousal dependent memory facilitation if such facilitation exists for spoken words. Additionally, happy prosody allowed us to determine whether the observed prosodic effect on word valence could be replicated for a positive emotion. If true, words with happy study prosody should induce more positive subsequent ratings than words with neutral study prosody.

### Experiment 2

#### Participants

Thirty-five undergraduate students participated in the experiment. Three participants were excluded from the data analysis. Two had a false alarm probability greater than 0.88 suggesting non-compliance with the task. One participant rated all word meanings with 0, suggesting exceptionally low emotion sensitivity or non-compliance with the task. Half of the remaining participants were female with an average age of 21 years (SD 0.8). Male participants were on average 21.44 years old (SD 1.3). Participants were enrolled in an introductory level psychology module and received course credit for participation. All participants reported normal or corrected to normal vision as well as normal hearing. They signed informed consent prior to the experiment.

#### Materials

The set of words selected for Experiment 1 was also used for Experiment 2. However, the words were spoken by a different female speaker. As for the first experiment, this speaker was selected based on her being best at conveying happiness and neutrality. The rating results for this speaker are presented in Table 1. The average duration of words spoken by her with a happy prosody was 680 ms (SD 98.7 ms) and that of words with a neutral prosody was 840.8 ms (SD 157).

#### Procedures

The procedures were identical to Experiment 1.

#### Results

**Figure 2:**
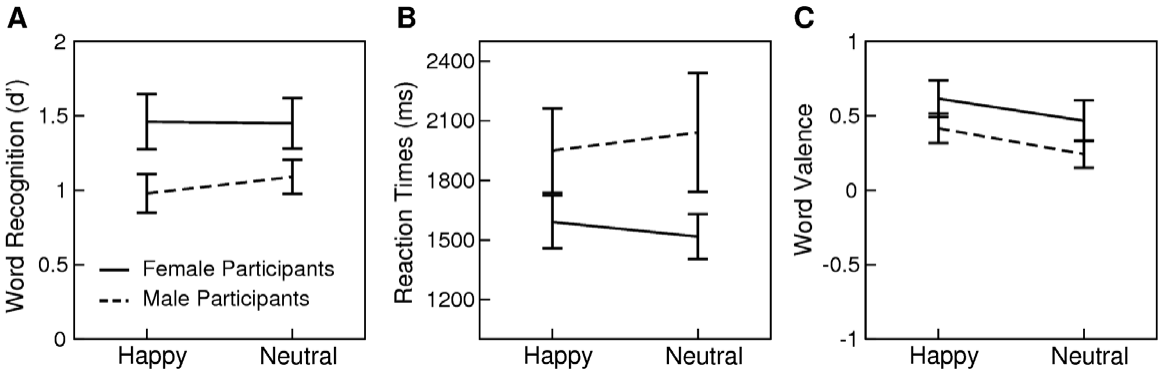
Results from Experiment 2. Mean d' scores and standard errors reflecting the sensitivity of discriminating old from new words are illustrated in graph A. Mean reaction times to correctly recognized old words are illustrated in graph B. Mean valence ratings of correctly recognized old words are illustrated in graph C.

The results of Experiment 2 are illustrated in Figure 2. The uncorrected probability of recognizing an old word as old was 0.74 (SD 0.15) for happy prosody and 0.73 (SD 0.15) for neutral prosody. Discrimination sensitivity as a function of study prosody was again assessed by computing d' scores and subjecting these scores to an ANOVA with *Prosody* as a repeated measures factor and *Sex* as a between subjects factor. With all other effects being non-significant (ps>.2), a marginal main effect of *Sex* suggested better word recognition in female as compared to male participants (F(1,30) = 4.13, p = .051). Again an ANOVA for reaction times was non-significant (ps>.12).

The effect of speaker prosody on word valence was assessed by subjecting the valence ratings of correctly recognized old words to an ANOVA with *Prosody* as a repeated measures factor and *Sex* as a between subjects factor. This analysis revealed a main effect of *Prosody* (F(1,30) = 4.89, p<.05) indicating that participants evaluated words as more positive, if these words had been spoken with happy (mean 0.52, SD 0.45) as compared to neutral prosody during study (mean 0.35, SD 0.47).

#### Discussion

The results of Experiment 2 largely replicated those of Experiment 1. Prosody again failed to influence verbal memory, but significantly modulated word valence. Happy prosody resulted in more positive word valence ratings than neutral prosody. Together with the results from Experiment 1, this suggests that prosodic context modulates a word's affective representation in semantic memory. Positive and negative prosody increase and decrease the pleasantness associated with a given word, respectively.

This effect may arise at three different processing stages. First, it may be a reflection of stimulus encoding. Specifically, a perceived mismatch between word valence and speaker prosody during study may lead to an immediate adjustment of word valence. Second, it may be a reflection of memory consolidation. Here, the adjustment would not be immediate but result from consolidation processes that bind prosodic context and word information (for a review see [31]). As in the first case, however, the adjustment would be complete upon word retrieval and possibly independent from the listeners' ability to recollect study prosody. Finally, one may speculate that the influence of prosody on word valence arises during memory retrieval. Participants may remember prior prosodic context during word recognition and base their valence ratings on this memory. This could occur implicitly, without the participants being aware of it, or explicitly with participants consciously adjusting the valence ratings to accord with the remembered prosody. In either case, however, the word valence effect would depend on and therefore correlate with the participants' memory for prosody.

Experiment 3 investigated this issue. As in Experiments 1 and 2, participants were presented with emotionally and neutrally spoken words during study and asked to memorize these words for a later recognition test. During test, they again performed an old/new judgment for each word. However, following this judgment they were now asked to either rate word valence or to indicate whether a word's prosody during study was neutral or emotional. The secondary judgments were performed in separate blocks and recorded as a within-participant variable to allow for a correlation analysis. If prosody modulates word valence during memory encoding or consolidation, memory for prosody should be irrelevant and hence may not correlate with the word valence effect. If, however, prosody modulates word valence during memory retrieval, memory for prosody should positively predict this modulation.

### Experiment 3

#### Participants

Forty-eight undergraduate students participated in the experiment. Half the participants were female and on average of 21 years old (SD 1.9). Male participants were on average 22.2 years old (SD 1.5). Participants were enrolled in an introductory level psychology module and received course credits for participating. All participants reported normal or corrected to normal vision as well as normal hearing. They signed informed consent prior to the experiment.

#### Materials

The materials were identical to Experiment 1.

#### Procedure

Each participant completed two study phases each followed by one test phase. The instructions for both study phases were identical to Experiment 1 and 2. Participants were again asked to focus on the words and to remember the words for a later recognition test. Moreover, as in the preceding experiments, participants were instructed to make old/new judgments in both test phases. However, only in one test phase was this judgment followed by a word valence rating. In the other test phase, participants were asked to indicate for any word that was judged as “old” whether its study prosody was sad or neutral. These latter judgments were made by pressing one of two buttons on the response box.

As for the preceding experiments, word lists were created, which were rotated across conditions and participants such that across participants each word appeared equally often as old or new word, equally often with sad or neutral prosody, and equally often in the word valence and the prosody memory tasks. We also counterbalanced the order of tasks and the assignment of left and right response buttons to the old/new and sad/neutral judgments.

Prior to the experiment, participants were instructed to listen to the words in each study phase and informed that their memory for these words would be assessed in a subsequent word recognition test. In order to clear any doubts about the general procedure, participants performed a practice run composed of six study trials followed by 12 test trials using the dummy words from the stimulus recording. Test trials in this practice ran comprised old/new decisions only. Participants were informed about the word valence rating and the prosody memory task only when commencing the respective test block in the actual experiment.

#### Results

**Figure 3:**
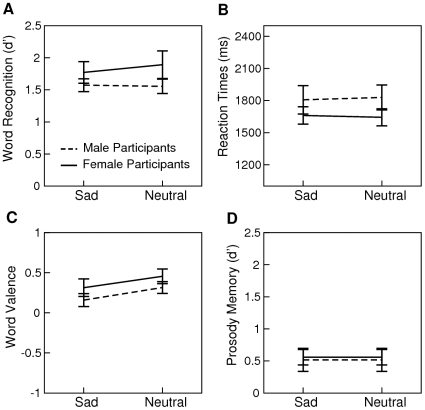
Results from Experiment 3. Mean d' scores and standard errors reflecting the sensitivity of discriminating old from new words are illustrated in graph A. Mean reaction times to correctly recognized old words are illustrated in graph B. Mean valence ratings of correctly recognized old words are illustrated in graph C. Mean d' scores reflecting the sensitivity of discriminating sad from neutral prosody for correctly recognized old words are illustrated in graph D.

The results from Experiment 3 are presented in Figure 3. The uncorrected probability of recognizing an old word as old was 0.68 (SD 0.18) for the emotional condition and 0.67 (SD 0.21) for the neutral condition. d' scores and reaction times were subjected to separate ANOVAs with *Prosody* as a repeated measures factor and *Sex* as a between subjects factor. Both analyses failed to reveal significant effects (ps>.16).

The influence of study prosody on a word's affective representation in semantic memory was assessed by subjecting the valence ratings of correctly recognized old words to an ANOVA with *Prosody* as a repeated measures factor and *Sex* as a between subjects factor. This analysis revealed a main effect of *Prosody* (F(1,44) = 6.56, p<.05) with the other main effect and interaction being non-significant (ps>.2). Thus, as in the two previous experiments, participants rated the valence of a word as more emotional if that word was presented with emotional (mean 0.24, SD 0.47) as compared to neutral prosody during study (mean 0.38, SD 0.41).

Participant's ability to accurately remember a word's study prosody was assessed by calculating a d' score. False alarms were identified as correctly recognized old words for which study prosody was incorrectly specified as sad. Hits were identified as correctly recognized old words for which study prosody was correctly specified as sad. The normalized probability of false alarms (i.e., number of false alarms divided by the number of correctly recognized old words with neutral study prosody) was subtracted from the normalized probability of hits (i.e., number of hits divided by the number of correctly recognized old words with sad study prosody). The obtained d' scores were relatively small (Mean 0.54, SD 0.74) but differed significantly from zero (t(47) = 5.02, p<.0001). Therefore, one can conclude that participants were better than chance in remembering study prosody.

**Figure 4:**
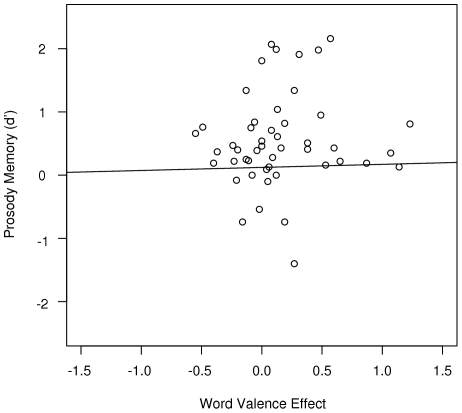
The relationship between memory for prosody and the word valence effect was non-significant.

Finally, we assessed whether conscious recollection of study prosody accounts for the observed word valence effect in two separate analyses. First, we subtracted mean valence ratings of correctly recognized words with sad study prosody from those with neutral study prosody. Across participants, this score was positive as words with sad study prosody tended to have a more negative rating than words with neutral study prosody. A one-tailed Pearson correlation analysis was used to test for a positive relationship between this score and the prosody memory d'. This analysis was non-significant (r = .09, p = .27, Figure 4) suggesting that participants' ability to recollect prosody does not predict whether and how prosody affects their affective representation of words in semantic memory. A second analysis was aimed at verifying that the word valence effect reported above would still be significant if inter-subject variation in prosody memory was entered into the model. To this end, an analysis with *Prosody* as a repeated measures factor, *Sex* as a between subjects factor, and *Prosody Memory d'* as a co-variate was performed. The *Prosody* main effect was again significant (F(1,44) = 6.47, p<.05).

#### Discussion

Experiment 3 replicates and extends the results of Experiments 1 and 2. Consistent with prior observations, the prosody effect on the speed and accuracy of verbal memory was non-significant reinforcing the idea that words are remembered equally well regardless of whether they are spoken with a neutral or an emotional prosody. Moreover, prosody again influenced word valence ratings indicating sustained prosodic effects on listeners. Analysis of prosody memory indicated that although participants were better than chance in remembering study prosody, their performance was nevertheless poor. Compared to the average d' for word recognition (mean 1.7, SD 1), the average d' associated with prosody recognition (mean 0.5, SD 0.7) was low. More importantly, however, the latter value failed to correlate with the word valence effect. Listeners who were good at remembering study prosody were not necessarily showing an influence of study prosody on word valence and vice versa. Thus, memory for prosody and the influence of prosody on word valence appear to be independent.

## Discussion

The present study investigated the influence of speaker prosody on the representation of verbal information in memory. Compared to neutrally spoken words, emotionally spoken words were expected to attract greater attention and to induce bodily arousal thereby enhancing memory for concurrent verbal information. Contrary to this expectation, however, word memory was comparable for neutrally and emotionally spoken words suggesting that prosody has little impact on memory storage of intentionally processed speech. This may be explained in several ways.

First, an effect of prosody on memory formation presupposes that listeners perceive the intended emotion state implicitly. Thus, one may question whether the prosodic manipulation used here was strong enough to enable such perception. While the word recognition results may suggest a lack of emotional strength, the word valence ratings speak to the contrary. Specifically, across three experiments, participants reliably discriminated between emotional and neutral study prosody. Moreover, this discrimination was evident during word recognition when no prosody information was provided and showed regardless of whether prosody was task-relevant. Hence, one can conclude that the emotions conveyed by prosody during study could be processed implicitly and should have been available for memory formation.

A second possible explanation for the failure of prosody to modulate verbal memory is that the emotions used here were inappropriate. To date, major evidence for an emotional facilitation of memory comes from studies that used threat related stimuli [26], [27], [29] raising the possibility that this facilitation is threat specific. However, some researchers identified memory facilitation for positive stimuli [28] providing evidence that such facilitation exists across emotion categories. Moreover, a recent verbal memory study conducted in our lab compared the effect of neutral and angry prosody and obtained similar results. If asked to remember a series of spoken words, participants' subsequent word recognition did not benefit from the prosodic threat context. Interestingly, a benefit emerged when participants were instructed to forget the studied words. Based on this and the present evidence, one can conclude that emotional prosody, regardless of valence and quality, leaves intentional memory storage unaffected but has sustained effects on existing memory representations by modulating their affective connotation.

That prosody fails to enhance intentional memory storage may be surprising. Comparable research using images, facial expressions, or words with affective or neutral connotations revealed relatively robust effects of emotion on memory [32]–[35]. However, such stimuli also reliably activate one of the key brain structures implicated in emotional processing - the amygdala [36]–[38]. In contrast, prosodic stimuli activate the amygdala less reliably. Most neuroimaging studies that compared emotional with neutral prosody in a whole brain analysis failed to identify amygdala contribution [39]–[44], [12], [21]. Moreover, when such a contribution was identified it typically involved a regions-of-interest approach [11], [9], [45] suggesting that the emotion evoked by prosody is not as strong as that evoked by other stimuli. Thus, prosody may fail to evoke sufficient bodily arousal to enable amygdala-dependent memory facilitation [25].

A potential reason for this is that prosodic emotional expression is constrained by language [46]. Emotions can only be conveyed to the extent that they allow speakers to articulate a verbal message. If emotional vocal modulations become too dominant they may interfere with linguistic production and communication may break down. Support for this argument comes from studies investigating non-linguistic vocalizations such as laughing or crying. Their emotional connotation is more accurately identified than that of speech prosody [46]. Moreover, like their facial analogues, these expressions reliably excite the amygdala and elicit bodily arousal [47]–[49] suggesting that vocalizations gain in emotional significance if they are freed from language. Moreover, like facial expressions or words, they may then be powerful enough to modulate memory storage.

Although the present study revealed no influence of prosody on verbal memory it nevertheless points to sustained prosodic effects on listener attitudes towards words and, by association, word referents. Words heard with a negative prosody assimilate negativity and words heard with a positive prosody assimilate positivity. Through this process, prosodic context moderates whether an individual will approach or avoid a word's referent in the future. Interestingly, this occurs independently from an individual's ability to remember prosody suggesting that prosodic moderation of word valence precedes word retrieval. Moreover, given that in two of the three experiments prosody was task-irrelevant it appears to be an implicit process.

Past research on the processing of prosody may offer insights into the mechanisms that underlie the observed valence effect. Specifically, work by Bach and colleagues [39] identified the amygdala and left STS as being particularly important for implicit prosodic processing. In their study, both structures were more strongly activated when participants categorized prosodic emotion as compared to when they categorized speaker sex. Moreover, these activations emerged when collapsing emotional and neutral prosody suggesting that they represent processes that are emotion-unspecific. In the amygdala these processes likely reflect relevance detection and the modulation of regions associated with stimulus processing [8], [44], [50]. In the STS these processes likely reflect higher order auditory functions such as the mapping of acoustic cues onto stored vocal representations with a particular significance to the individual [51], [52]. Additionally, through connections with other temporal and frontal lobe structures [53]–[56], both the amygdala and the STS communicate with regions involved in language processing. As such they may be critical in mediating the effects observed in the present study. For example, one could envision that vocal information represented in the STS is matched against verbal representations in regions posterior and inferior to the STS. In case of incongruity, the largely biologically determined vocal representations may shape the stored linguistic symbols.

Evidence in support for this speculation comes from functional neuroimaging research that identified greater activation for emotional words spoken with incongruous as compared to congruous emotional prosody. Positive and negative words spoken with a negative or positive prosody, respectively, were found to activate the inferior frontal gyrus [21], [22]–a structure implicated in word retrieval. Additional evidence comes from studies measuring event-related potentials (ERPs). These revealed a larger negativity around 400 ms following words with incongruous as compared to congruous emotional prosody (e.g., happily spoken “success”; [18], [57]). This is comparable to a negativity with frontal and temporal generators that is elicited for words presented in a semantically incongruous as compared to congruous sentence context [58], [59]. Importantly, the observed negativity is not only increased for complete incongruity but also for a partial incongruity as arising from a neutral word meaning and an emotional prosody [18], [57].

Based on this and the present results, one may speculate that in addition to modulating word retrieval, incongruity between prosody and word meaning triggers processes that calibrate linguistic representations to better map onto accompanying vocal context. Future research involving online measures of neural processing will be necessary to validate this hypothesis and contrast it with a potential modulation occurring after stimulus processing. Rather than stimulus encoding, it is possible that prosodic modulation of word valence occurs during memory consolidation where content and context are bound to enable integrative event memories (for a review see [31]).

Taken together, the present results extend the existing literature by highlighting sustained changes in verbal representations as a function of speaker prosody. As such they point to a mechanism by which words - in the course of repeated interactions and through the integration with other contextual cues - acquire an emotional significance that may be salient enough to excite automatic appraisal and lead to bodily arousal [36]. The functionality of such a mechanism is easy to conceive. Among others, it would allow individuals to acquire adequate emotional responses, not just to a word's referent, but to the word itself allowing the word to effectively guide behaviour. This notion is in line with observations of language learning in childhood. Such observations revealed that adults use a different mode of speech when interacting with infants and young children as compared to adults. This mode, termed infant-directed (ID) speech, is produced at a higher pitch and with greater prosodic variation than the so called adult-directed (AD) speech. Researchers have proposed that ID speech serves attentional engagement [60] and language learning by allowing infants to identify important units of speech [61]–[63]. Additionally, ID speech has been implicated in emotional communication. Research by Trainor and colleagues [64] revealed strong similarities between ID speech and emotionally expressive speech directed at adults. The authors, therefore, proposed that ID speech promotes emotional exchanges and bonding with the infant. The present results extend this idea. ID speech conveys not only relational emotional information but emotional information about communication referents. The child can thus learn which emotions correspond to which objects or events in the environment and link these emotions to the accompanying words. As for the adult participants tested here, these words then acquire a valence that informs subsequent behaviour.

While providing intriguing evidence for sustained effects of speaker prosody on listeners, the present results should nevertheless be viewed preliminary. To better understand the modulation of stored word valence by speaker prosody, one may wish to examine the relationship between memory for prosody and word valence within a participant and within a given item. This was not possible here as different items were presented in the different tasks. Participants performed the word valence judgment on a different set of words than the prosody memory task. The rational for this was that if asked to remember prosody and judge word valence for the same item, participants would potentially confound the two. Future research could address this issue by using the same stimuli in a word valence task and a prosody memory task but separating them by several days. Alternatively, one could measure neuronal activity during initial and subsequent encounters with a word. This might allow the identification of encoding processes that predict later changes in word valence.

To conclude, the present study found speaker prosody to be irrelevant for subsequent word recognition but important for shaping a word's affective representation in memory. Words produced with an emotional tone assimilate that tone thereby becoming more emotional themselves. Given that this occurs without intention and independently of memory for prosody, one can infer this process to be automatically triggered during speech processing. Through this, speakers can produce attitude changes in their listeners that outlast the moment and that allow their message to have a long-term influence on listener behaviour.
